# Carotid approach to anterior circulation thromboembolectomy in an adult with failing fontan physiology: a case report

**DOI:** 10.1186/s12871-021-01364-z

**Published:** 2021-05-18

**Authors:** Caroline Eden, Hugo Clifford, Arthur Wang, Asif Mohammed, Peter Yim

**Affiliations:** 1 Department of Anesthesiology, Columbia University College of Physicians and Surgeons, NY New York, USA; 2 Department of Anesthesiology, New York-Presbyterian, Columbia University Medical Center, 622 W 168th St, NY 10032 New York, USA; 3 Department of Neurological Surgery, Columbia University College of Physicians and Surgeons, NY New York, USA; 4 Department of Anesthesiology, Perioperative Medicine and Pain Management, University of Miami, Miller School of Medicine, Florida Miami, USA

**Keywords:** Fontan Procedure, Congenital Heart Disease, Thromboembolism, Interventional Radiology

## Abstract

**Background:**

Anesthetic management of an adult with failing Fontan physiology is complicated given inherent anatomical and physiological alterations. Neurosurgical interventions including thromboembolectomy may be particularly challenging given importance of blood pressure control and cerebral perfusion.

**Case Presentation:**

We describe a 29 year old patient born with double outlet right ventricle (DORV) with mitral valve atresia who after multi-staged surgeries earlier in life, presented with failing Fontan physiology. She was admitted to the hospital almost 29 years after her initial surgeries to undergo workup for a dual heart and liver transplant in the context of a failing Fontan with elevated end diastolic pressures, NYHA III heart failure symptoms, and liver cirrhosis from congestive hepatopathy. During the workup in the context of holding anticoagulation for invasive procedures, she developed a middle cerebral artery (MCA) stroke requiring a thromboembolectomy via left carotid artery approach.

**Discussion and Conclusions:**

This case posed many challenges to the anesthesiologist including airway control, hemodynamic and cardiopulmonary monitoring, evaluation of perfusion, vascular access, and management of anticoagulation in an adult patient in heart and liver failure with Fontan physiology undergoing thromboembolectomy for MCA embolic stroke.

## Background

The anesthetic management of an adult with failing Fontan circulation, especially one with multiorgan failure, and known Fontan thrombus is extremely complex. As Rychik notes, “management of even simple medical problems may be complicated by the hemodynamic deficiencies of the Fontan circulation” [1]. Furthermore, carotid access for thrombectomy is rare; one case series showed this access was necessary in 7 patients out of 151 who received endovascular thrombectomy [2]. In addition, it has not been described in an adult patient with failing Fontan physiology and poor vascular access. In this patient, with failing Fontan physiology, there were various aspects of the anesthetic and surgical approach to middle cerebral artery (MCA) thrombectomy that were challenging. The purpose of this case report is to examine the perioperative management and difficulties in an acutely decompensating patient with failing Fontan physiology including control of the airway, perfusion, vascular access, and hemodynamic monitoring. Written informed consent for publication of this report was obtained from the patient’s healthcare proxy. HIPAA authorization has been obtained.

## Case Presentation

We describe a 29 year old woman (45.4 kg, 155 cm, BMI 18.9) born with double outlet right ventricle (DORV) with mitral valve atresia, admitted to the hospital for workup for a heart and liver transplant in the context of elevated end diastolic pressures, heart failure symptoms, and liver cirrhosis from congestive hepatopathy in the context of a failing Fontan. Her course was complicated by middle cerebral artery (MCA) thrombus requiring cerebral angiography and mechanical thrombectomy.

Surgical history is notable for modified fenestrated lateral tunnel Fontan with atrial septectomy at age 2 for repair of her congenital anomaly, that was complicated by a Fontan thrombus requiring a bidirectional Glenn procedure and subsequent redo fenestrated lateral tunnel Fontan with tricuspid valve annuloplasty.

In late 2018, she developed symptoms concerning for Fontan failure, and underwent a cardiac catheterization, which revealed elevated end diastolic and Fontan pressures, and cardiac output of 3.5 liters per minute. Her baseline oxygen saturation during this time was noted as “high 70s-low 80s on room air”, blood pressure 106/64 mmHg, heart rate 80 beats per minute, and a respiratory rate of 14 breaths per minute A Computed Tomography (CT) abdomen and pelvis showed liver cirrhosis and selected liver function labs were as follows: INR 3.08, PT 31.8, Total Bilirubin 3.1, and Direct Bilirubin 1.2. Initial workup was complicated by thalamic ischemic strokes while holding home warfarin; she recovered without neurological deficits.

### On Admission

In July 2019, she was admitted to the hospital for workup of heart and liver transplantation in the context of failing Fontan circulation. On admission she was alert and oriented, and edematous in her abdomen and legs bilaterally; vital signs were notable for an oxygen saturation of 78 % on 4 L nasal canula. Per outpatient notes, her baseline oxygen saturation was 70 s to low 80 s of room air, with oxygen requirement at night. She was bridged from warfarin to heparin in anticipation of a transesophageal echocardiogram (TEE) and liver biopsy. Heparin was held for four hours, two hours before and after the procedure. The TEE showed filamentous material and spontaneous echogenic contrast swirling at the junction of the Fontan and the main pulmonary artery. Several hours following the TEE, the patient developed altered mental status, vomiting, hypotension to 50 s/30s mm Hg, and oxygen saturations to 70 %. She was deemed unable to protect her airway and the Anesthesiology team was called for emergent intubation.

### During Decompensation

Positive pressure mask ventilation was attempted despite aspiration risk with continued desaturation; she was subsequently induced with 140 mg succinylcholine, 40 mg propofol, and 20 mcg epinephrine. Direct laryngoscopy with macgrath 3 (video laryngoscope) revealed grade 1 view; however, the endotracheal tube could not be passed given an anterior airway. She was ventilated via a face mask, on 50 mg rocuronium, and on second attempt the 7.0 endotracheal tube was passed atraumatically through the vocal cords. To maintain the hemodynamic status during induction into anesthesia and transfer to mechanical ventilation, a total bolus of 250 µg of epinephrine was administered. After intubation, analgosedation was performed using a continuous infusion of fentanyl at 25 mcg/hr, midazolam at 5 mg/hr. Correction and maintenance of hemodynamics was performed by continuous infusion of dopamine 10 mcg/kg/hr and phenylephrine 400 mcg/kg/min.

Given concern for thrombotic versus hemorrhagic stroke, heparin, which had been restarted upon returning to the floor, was held. She was stabilized, and taken for CT head/CT Angiogram (CTH/CTA) head and neck for stroke workup. At the time, PTT < 40 and INR was 1.5. Intravenous tissue plasminogen activator (IV-tPA) was administered given concern for a large intracranial vessel occlusion. CTA demonstrated a left MCA occlusion and the patient was brought emergently to the neurointerventional suite for emergent mechanical thrombectomy. She arrived with a 20 g IV in the left antecubital fossa, and a triple lumen central line in the left femoral vein. A left radial arterial line was placed.

She was placed on a ventilator with settings as follows: pressure control, peak inspiratory pressures between 20 and 25 cm H2O, PEEP between 1 and 2 cm H2O, respiratory rate at 14 breaths per minute, with estimated tidal volumes around 340mLs. Her vitals were as follows: blood pressure 112/82 mmHg, heart rate 102 beats per minute, and oxygen saturation of 74.6 %. Her ETCO2 averaged 20 mm Hg presumably given V/Q mismatching from low perfusion of the lungs. Arterial access for the angiogram was difficult given prior history of cardiac catheterizations and known right common femoral artery occlusion. Ultrasonographic guidance was used in an effort to obtain right common femoral, then left common femoral arterial access. After failed attempts at transfemoral access, the left common carotid artery was accessed using 2 dimensional and color Doppler sonographic guidance and an 18-gauge hollow core needle. Use of the radial artery catheterization was attempted and deferred given the patient’s small habitus and difficulties in placing a larger gauge arterial line. Mechanical thrombectomy using a stent was successfully performed and the left MCA was revascularized. Both femoral and carotid introducers were left in place given recent administration of tPA.

### After Intervention

The patient was then transferred to the Neurosurgical Intensive Care Unit. Her vital signs at the time were blood pressure 114/92 mmHg, 92 beats per minute, 23 respirations per minute, and oxygen saturation of 84 %. Her arterial blood gas on arrival was as follows: pH 7.18, paO2 50 mmHg, PCO2 47 mmHg, bicarbonate 15 mEq/L. Given the stroke burden, the team determined she would most likely be dependent on others long term, unable to walk and talk. Her family determined she would not consider this quality of life and she was made comfort care two days following the procedure. She passed away the following day.

## Discussion and Conclusion

Thrombosis and anticoagulation is a critical consideration in patients with Fontan circulation for a few reasons. Fontan circulation even when it is *functioning* is the ideal substrate for Virchow’s triad with endothelial damage given changes in systemic pressures, distinct states of altered blood flow, and intrinsic plasma protein changes given liver disease and protein losing enteropathy [3]. Polycythemia from chronically low oxygen saturations is common and leads to increased blood viscosity predisposing to thromboembolic events [4]. One retrospective study showed an overall occurrence rate of 3.9 events per 100 patient-years, with an overall mortality rate of 21 % in those with a thrombus [5]. One study showed that Fontan patients on an antiplatelet or anticoagulant had lower rates of death compared to those who were not [6, 7]. Given the risk of thromboembolic events in patients with Fontan circulations and evidence that aspirin may reduce intracardiac thrombus in Fontan patients, it is perhaps reasonable to start aspirin, in the absence of systemic anticoagulation [8].

Intraoperatively, there were difficulties in gaining femoral arterial access for cerebral angiography and thrombectomy. This was likely complicated by prior cardiac catheterizations presumably leading in part to her right femoral clot and arterial wall damage, differentiating venous from arterial circulation given physiological and pathological shunting as well as altered flow dynamics of the arterial blood, and her non-anatomical vascular landmarks. The utility of carotid artery access is limited. Indications include critical aortic stenosis and relief of occlusions from aorto-pulmonary collaterals [9]. One author remarks: “transfemoral catheterization is likely to be challenging, a low threshold for considering whether switching to the carotid approach could be of therapeutic benefit” [2]. Indeed, it may be appropriate to consider initial carotid artery approach for anterior circulation thrombectomy in adults with congenital heart disease who have undergone extensive repair.

If extracorporeal membrane oxygenation (ECMO) is anticipated, site of cannulation should be consideration as blind cannulation past the hepatic inferior vena cava could lead to unintentional access into the pulmonary artery or the fenestrated Fontan.

Assessment of brain perfusion and hemodynamics is particularly complex in a stroke patient undergoing cerebral angiography and potential thromboembolectomy. According to the Society for Neuroscience in Anesthesiology and Critical Care, systolic blood pressure should be maintained > 140 mm Hg (fluids and vasopressors) and < 180 mm Hg and diastolic blood pressure < 105 mm Hg (class IIa, level of evidence B) [10]. However, there is still conflicting evidence guiding blood pressure management in ischemic stroke within the first twelve hours after onset, it is physiologically reasonable to “avoid blood pressure lowering medications” [11]. Overall, it is critical to obtain baseline values including cardiovascular history, baseline blood pressure (prior to admission and at admission), oxygen saturation, pulmonary vascular resistance via review of heart catheterization and priori mental status to guide the complex decisions underlying hemodynamic managements in patients undergoing cerebral angiography.

Another consideration in flow dynamics in patients with failing Fontan physiology is the assessment of the pulmonary vascular resistance (PVR). It is critical to maintain a low PVR by avoiding hypercarbia, hypoxia, hypothermia and pain and considering inhaled nitric oxide in Fontan patients given their dependence on passive blood flow through the lungs for blood oxygenation.

Cardiac output and systemic vascular resistance may not be reflective of tissue of perfusion, especially in a patient with failing Fontan physiology dependent on low systemic and pulmonary vascular resistance for forward flow. Direct measurement of cerebral perfusion may be useful in guiding hemodynamic management. In our patient, who had a baseline oxygen saturation around 80 % and was cyanotic at the time of admission, how can we determine what level of oxygen saturation is adequate for brain perfusion? Cooximetry, a device that uses spectrophotometry to measure relative blood concentrations of various forms of hemoglobin, may be one method to more accurately determine concentrations of oxygen in the blood. This may be especially useful in a patient with failing Fontan physiology with largely decreased peripheral perfusion and different degrees of shunting throughout the body. Near-infrared spectroscopy (NIRS), a non-invasive monitor used to monitor cerebral oxygenation, may also be useful in examining any changes in baseline cerebral perfusion with changes in blood pressure or potential neurosurgical intervention. Given injection of dye during angiography, the output may not be accurate, but this effect may be shortlived [12]. In a hemodynamically unstable adult patient with failing Fontan physiology, advanced monitoring for global perfusion may be beneficial.

Perioperative management of patients with failing Fontan and known Fontan thrombus should aim to expediently restart anticoagulation and begin aspirin if feasible, assess vascular access and anatomy, consider alternate devices to measure cerebral perfusion, and evaluate each individual patients’ hemodynamic baselines.

## Data Availability

There is no data to be shared.

## Abbreviations

DORV: Double outlet right ventricle
MCA: Middle cerebral artery
CT: Computed Tomography
TEE: Transesophageal echocardiogram
CTH/CTA: CT head/CT Angiogram
IV-tPA: Intravenous Tissue plasminogen activator
ECMO: Extracorporeal membrane oxygenation
PVR: Pulmonary vascular resistance
